# Atypical Hippo signaling network: uncovering novel insights into head and neck cancer biology and advancements in precision intervention

**DOI:** 10.3389/fcell.2025.1610471

**Published:** 2025-05-23

**Authors:** Pengfei Yang, Shujuan Li

**Affiliations:** ^1^ Emergency Department Outpatient Chemotherapy Center, Yunnan Cancer Hospital, The Third Affiliated Hospital of Kunming Medical University, Peking University Cancer Hospital Yunnan, Kunming, Yunnan, China; ^2^ Department of Nuclear Medicine, Yunnan Cancer Hospital, The Third Affiliated of Kunming Medical University, Peking University Cancer Hospital Yunnan, Kunming, Yunnan, China

**Keywords:** atypical Hippo signaling, head and neck cancer, kinase, multipathway crosstalk, PTM homeostasis

## Abstract

As a major global health challenge with rising incidence and poor prognosis, head and continues to impose a significant clinical burden due to its aggressive biological behavior and frequent therapeutic resistance. Within this context, the atypical Hippo signaling pathway emerges as a crucial regulatory network, integrating diverse components including core kinases (TAO kinases, MAP4K family, NDR1/2 kinases), cell polarity determinants (CRUMBS, SCRIBBLE), junctional adhesion molecules (AMOT family), phosphorylation mediators (14-3-3 proteins), and tumor suppressors (NF2, RASSF family). This multifaceted system governs fundamental cellular processes spanning proliferation, apoptosis, migratory capacity, and immune microenvironment modulation. Notably, post-translational modifications (ubiquitination, acetylation, SUMOylation) of pathway components dynamically regulate the stability and activity of downstream effectors YAP/TAZ, whose sustained activation through molecular aberrations drives tumor progression and treatment resistance in head and neck malignancies.The pathway’s extensive crosstalk with Wnt signaling, NF-κB cascades, and estrogen receptor networks creates context-dependent regulatory plasticity that contributes to tumor heterogeneity. Current therapeutic innovation focuses on molecular diagnostics and precision targeting approaches, including direct YAP/TAZ-TEAD complex inhibitors, upstream receptor modulators, and rational combinations with immune checkpoint blockade. Future investigations should employ multi-omics profiling to delineate tumor subtype-specific regulatory architectures while advancing novel drug delivery platforms. These efforts promise to translate mechanistic insights into multi-targeted therapeutic strategies capable of overcoming resistance mechanisms and improving survival outcomes for this therapeutically challenging malignancy.

## 1 Introduction

Head and neck cancer is a prevalent malignancy with poor prognosis worldwide, posing significant clinical challenges due to its anatomical complexity and difficulties in early diagnosis (Chowl, 2020). In recent years, with continuous advancements in molecular biology research, the Hippo signaling pathway has garnered substantial attention for its critical roles in regulating organ size, tissue regeneration, and tumorigenesis (Moya and halder, 2019; Dey et al., 2020). The classical Hippo pathway primarily relies on the cascade reactions of MST1/2 and LATS1/2 kinases, which restrict cell proliferation by inhibiting the nuclear translocation of the transcriptional co-activators YAP/TAZ. However, recent studies have revealed that, in addition to the classical pathway, the non-classical Hippo pathway also modulates cell fate through a complex signaling network, with its regulatory components and mechanisms exhibiting remarkable diversity and hierarchical organization.

The non-classical Hippo pathway not only comprises core kinase components—such as TAO kinases (TAOK1/2/3) (Fang et al., 2020), MAP4K family kinases (Chen et al., 2019), and NDR1/2 kinases (Ye et al., 2020)—which regulate cell proliferation, apoptosis, and migration via pathways independent of the classical MST1/2-LATS1/2 axis, but also involves a series of proteins closely associated with cell polarity, junctions, and adhesion, including CRUMBS (Thompson et al., 2013), SCRIBBLE (Buckley and st johnston, 2022), and AMOT family proteins (Wang et al., 2022). In addition, the 14-3-3 protein, serving as a critical phosphorylation adaptor, plays an essential role in maintaining intracellular protein localization and signal transduction (Hermann et al., 2021). Simultaneously, tumor suppressors such as NF2 (Merlin) and members of the RASSF family establish a defensive barrier for the non-classical Hippo pathway by modulating upstream kinase activities and stabilizing the cytoskeleton (Fu et al., 2022; Djos et al., 2012). Furthermore, post-translational modifications of proteins in the non-classical Hippo pathway—such as the ubiquitination, acetylation, and SUMOylation of MST1/2, ΔNp63, TEAD family members, RASSF1A, Beclin 1, and MOB proteins—significantly affect downstream signaling and YAP/TAZ activity, thereby playing crucial roles in the regulation of cell proliferation, apoptosis, and autophagy, and are closely linked to drug resistance and invasiveness in head and neck cancer (Sannigrahi et al., 2015; Lin et al., 2024; Fang et al., 2018). More complex still, extensive crosstalk exists between the non-classical Hippo pathway and the Wnt, NF-κB, and estrogen receptor (ER) signaling pathways; these interactive networks jointly regulate cell fate, immune responses, metabolic reprogramming, and the formation of the tumor microenvironment (Zhao et al., 2023; Britschgi et al., 2017; Ma et al., 2021). For example, VGLL4, a downstream transcriptional co-regulator within the non-classical Hippo pathway, competitively binds TEAD, thereby not only inhibiting YAP/TAZ-mediated gene expression but also synergistically suppressing Wnt/β-catenin signaling, which in turn impedes tumor cell proliferation and metastasis (Jiao et al., 2017). Additionally, the interplay between the NF-κB pathway and ER signaling with the Hippo pathway further influences the biological behavior and drug resistance of head and neck cancer, representing a promising breakthrough for future precision therapies (Ma et al., 2021).

This review aims to systematically examine the various components of the non-classical Hippo pathway and their post-translational regulatory mechanisms, as well as to explore the crosstalk between this pathway and other signaling pathways, including Wnt, NF-κB, and ER, in the context of head and neck cancer development. By comprehensively analyzing current research progress, we hope to provide new theoretical insights and practical guidance for early diagnosis, prognostic assessment, and precision treatment of head and neck cancer.

## 2 Overview of the Hippo signaling pathway

### 2.1 Overview of the classical Hippo pathway

The classical Hippo signaling pathway is primarily composed of a highly conserved kinase cascade, with core components including the MST1/2 kinases, their adaptor protein SAV1, the downstream LATS1/2 kinases, and the regulatory protein MOB1 (Wu et al., 2003). This cascade transmits signals via sequential phosphorylation: MST1/2 kinases activate SAV1 and MOB1, which in turn activate the LATS1/2 kinases (Huang et al., 2005). Once activated, LATS1/2 phosphorylate the transcriptional coactivators YAP and TAZ, inducing conformational changes that expose masked nuclear localization signals (NLS), thereby promoting their cytoplasmic retention or leading to subsequent ubiquitination and degradation (Dong et al., 2007) (Supplementary Figure S1). Consequently, the classical Hippo pathway plays a critical inhibitory role in cell proliferation, apoptosis, and tissue size control, with its dysregulation often closely associated with tumorigenesis and cancer progression.

### 2.2 Definition and characteristics of the non-classical Hippo pathway

The non-classical Hippo pathway refers to regulatory mechanisms that modulate cell fate independently of the traditional MST1/2–LATS1/2 kinase axis. These pathways exhibit a more diversified regulatory approach with the participation of alternative signaling cascades, incorporating factors such as mechanical stress, inflammatory signals, metabolic status, and other extracellular environmental changes. For example, under certain circumstances, cells can directly regulate the subcellular localization of YAP/TAZ via mechanical tension or cell-contact signals without engaging the complete MST1/2–LATS1/2 cascade (Cho and Jiang, 2021). Additionally, inflammatory mediators such as TNF-α or the NF-κB pathway can modulate the output of the Hippo pathway through cross-regulatory mechanisms (Wang et al., 2020). This non-classical mode of regulation reflects the complexity and multi-layered nature of the signaling network, offering enhanced flexibility for cellular adaptation to a dynamic environment, while also providing new insights into the mechanisms of signal dysregulation during tumorigenesis (Ma et al., 2021; Chen, 2019).

## 3 The relationship between non-classical Hippo pathway components and head and neck cancer

**FIGURE 1:**
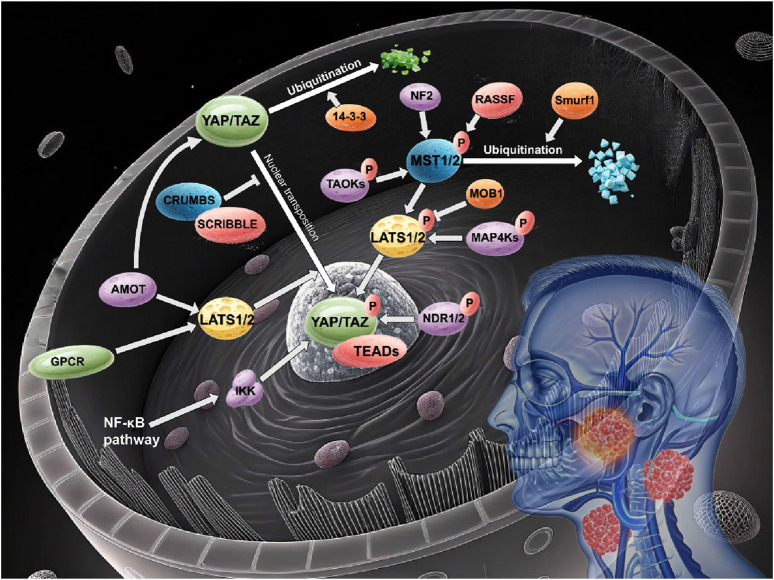
Non-canonical hippo pathway in head and neck cancer.

The non-classical Hippo signaling pathway has emerged as a critical contributor to the pathogenesis of head and neck cancer, with its distinct regulatory components and complex cross-talk mechanisms influencing tumor proliferation, invasion, and chemoresistance (Figure 1).

### 3.1 Non-classical Hippo pathway core kinase components

#### 3.1.1 TAO kinases (TAOK1/2/3)

TAO kinases (TAOK1/2/3), belonging to the STE20 kinase family, serve as crucial upstream regulators of the Hippo signaling pathway. Current studies indicate that TAO kinases can phosphorylate and activate MST1/2, thereby indirectly promoting LATS1/2 activation. This cascade leads to the phosphorylation of YAP/TAZ, resulting in conformational changes that expose masked nuclear localization signals (NLS), thus causing cytoplasmic retention or ubiquitin-mediated degradation of YAP/TAZ (Fang et al., 2020; Kapfhamer et al., 2012; Boggiano et al., 2011). Additionally, some studies suggest that TAO kinases (e.g., TAOK3) may further enhance LATS1/2 kinase activity by directly phosphorylating hydrophobic motifs within LATS1/2 (Fang et al., 2020; Boggiano et al., 2011). This regulatory mechanism is essential for maintaining proper cell proliferation, apoptosis, and tissue homeostasis, while also significantly impacting tumorigenesis.

It has been reported that head and neck squamous cell carcinoma samples often exhibit high expression levels of TAO kinase family members. This aberrant expression may reduce LATS1/2 activity or disrupt the normal phosphorylation cascade, leading to persistent dephosphorylation and excessive nuclear localization of YAP/TAZ, which in turn activates downstream pro-growth and pro-invasive target genes (Faraji et al., 2022; Shin and Kim, 2020). For example, the EGFR signaling pathway is frequently abnormally active in head and neck cancer; its effects may be partly mediated by interfering with MOB1-dependent LATS1/2 activation in synergy with TAO kinase upregulation, collectively promoting YAP/TAZ activation (Ando et al., 2021). Moreover, in other tumor types such as lung, breast, and colon cancers, TAO kinases along with MAP4K family kinases have been shown to exhibit certain tumor-specific roles. Notably, high TAOK3 expression in breast cancer has been associated with the HER2-positive subtype, potentially facilitating tumor metastasis through activation of the PI3K/AKT signaling pathway (Boggiano et al., 2011).

#### 3.1.2 MAP4K family kinases

MAP4K family kinases are also pivotal components of the Hippo signaling pathway. Research has demonstrated that MAP4K family members (e.g., MAP4K1/2/3 and MAP4K4/6/7) can cooperate with MST1/2 by directly phosphorylating key residues on LATS1/2—such as hydrophobic motifs and activation loops—thus promoting LATS1/2 autophosphorylation and full activation (Meng et al., 2015; Li et al., 2015). This redundant and complementary activation mechanism ensures the stringent regulation of YAP/TAZ under various cellular conditions. In addition, MAP4Ks may also influence YAP/TAZ stability and subcellular localization via non-classical pathways (e.g., through modulation of AMPK or mTOR signaling) (Meng et al., 2015; Gao et al., 2016).

The role of MAP4K family kinases in head and neck cancer is gradually gaining attention. Some studies have indicated that members such as MAP4K4 in head and neck cancer may enhance tumor cell migration and chemoresistance by activating the JNK signaling pathway (Meng et al., 2015; Li et al., 2015). Furthermore, in oral squamous cell carcinoma, downregulation of LATS1/2 expression is closely associated with enhanced nuclear localization of YAP/TAZ, and MAP4K family kinases may act as alternative kinases in this context, maintaining partial Hippo signaling that affects tumor stem cell self-renewal and metastasis (Faraji et al., 2022). In other tumor types, MAP4K4 has been implicated in promoting tumor cell invasion and resistance in pancreatic cancer and melanoma through activation of the JNK and RhoA signaling pathways (Li et al., 2015; Gao et al., 2016).

#### 3.1.3 NDR1/2 kinases

NDR1/2 kinases belong to the NDR/LATS kinase family. In the classical Hippo signaling pathway, MST1/2 kinases phosphorylate and activate LATS1/2, which in turn maintain YAP/TAZ in a phosphorylated state, thereby preventing their nuclear translocation and subsequent pro-proliferative effects. However, recent studies have revealed that NDR1/2 kinases not only contribute to classical signal transduction but also play key roles in the non-classical regulation of the Hippo pathway.

In non-classical regulatory mechanisms, NDR1/2 kinases can act in concert with upstream kinases (such as MST1/2) or, in certain contexts, independently regulate the phosphorylation status of YAP/TAZ. For example, by directly phosphorylating specific residues on YAP/TAZ, NDR1/2 can modulate their subcellular localization and transcriptional activity, thereby influencing cell proliferation, apoptosis, and cell cycle progression (Hergovich, 2016; Hergovich, 2013). In addition, NDR1/2 play important roles in cell cycle regulation by modulating the stability of cell cycle inhibitors (such as p21), thus affecting the G1/S transition and maintaining cellular growth homeostasis (Cornils et al., 2011).

Studies on solid tumors have shown that NDR1/2 kinases exhibit complex functions. For instance, in glioblastoma, NDR1 has been reported to phosphorylate YAP, thereby suppressing its oncogenic activity and exerting a tumor-suppressive effect (Chen et al., 2021); in colorectal cancer, loss of NDR1/2 correlates with YAP dephosphorylation, leading to uncontrolled cell proliferation and carcinogenesis (Zhang et al., 2015); in prostate cancer, NDR1 appears to inhibit tumor metastasis through crosstalk with the Wnt pathway (Xuan et al., 2024), although its impact on tumor immunity manifests as a pro-tumorigenic effect (Fu et al., 2024). Although direct studies on NDR1/2 in head and neck cancer are relatively limited, research has implicated the loss of the tumor suppressor NDRG2 in the development of oral squamous cell carcinoma (OSCC) via modulation of PI3K/AKT-mediated dephosphorylation of PTEN at S380/S382/T383 (STT) (Tamura et al., 2017). Given that head and neck squamous cell carcinoma is often characterized by aberrant activation of YAP/TAZ, NDR1/2 kinases may also have significant regulatory roles in this tumor type, where their dysregulation could disrupt Hippo signaling and thereby promote tumor cell proliferation, invasion, and drug resistance.

### 3.2 Non-classical Hippo pathway cell polarity components

Cell polarity and adhesion proteins are fundamental to maintaining normal epithelial cell functions and are essential prerequisites for proper activation of the Hippo signaling pathway. Polarity proteins such as CRUMBS and SCRIBBLE not only determine the apical-basal polarity of cells but also regulate membrane architecture and cell–cell adhesion, indirectly restricting the nuclear translocation of YAP/TAZ and thereby preventing excessive cell proliferation and abnormal tissue growth.

#### 3.2.1 CRUMBS protein

CRUMBS proteins form a complex with intracellular partners (such as Pals1 and Patj) to establish and maintain the apical polarity of cells (Muthuswamy and Xue, 2012; Stephens et al., 2018). The proper localization of this complex is instrumental in anchoring the adaptor protein Expanded (Ex) at the apical membrane, thereby facilitating activation of the Hippo signaling cascade (MST1/2→LATS1/2) which leads to the phosphorylation and cytoplasmic retention of YAP/TAZ (Bulgakova and Knust, 2009; Eaton and Martin-belmonte, 2014). Studies have demonstrated that loss or downregulation of CRUMBS proteins results in mislocalization of Ex, insufficient LATS1/2 kinase activity, and consequent excessive dephosphorylation and nuclear accumulation of YAP/TAZ. This aberrant activation triggers pro-proliferative and anti-apoptotic gene expression, promoting tumorigenesis (Huang and Muthuswamy, 2010). In epithelial-derived tumors such as head and neck cancer, downregulation of CRUMBS family members (e.g., CRB3) is closely associated with loss of cell polarity, epithelial–mesenchymal transition (EMT), and aberrant activation of YAP/TAZ, potentially driving tumor invasion and metastasis (Faraji et al., 2022; Chen et al., 2010).

#### 3.2.2 SCRIBBLE protein

SCRIBBLE is another critical regulator of cell polarity that, together with Dlg and Lgl in a multi-protein complex, maintains basal polarity and overall structural integrity of cells (Margolis, 2018; Kumichel and Knust, 2014). In addition to stabilizing epithelial cells via regulation of cell–cell adhesion (e.g., through interaction with E-cadherin), SCRIBBLE can interact directly or indirectly with core components of the Hippo pathway (such as MST1/2 and LATS1/2) to promote the phosphorylation and cytoplasmic retention of YAP/TAZ (Charrier et al., 2015). In head and neck cancer, particularly in HPV-associated head and neck squamous cell carcinoma, the HPV E6 protein frequently targets SCRIBBLE through its PDZ-binding motif, leading to its ubiquitination, degradation, and functional loss (Bazzoun et al., 2013). Loss of SCRIBBLE function disrupts cell polarity balance, facilitating the nuclear entry of YAP/TAZ, which subsequently activates downstream pro-tumorigenic transcriptional programs and promotes tumor growth and invasion (Kumichel and Knust, 2014; Bazzoun et al., 2013; Zhan et al., 2008).

### 3.3 Non-classical Hippo pathway cell junction and adhesion protein components

The Angiomotin (AMOT) protein family comprises cell junction-associated proteins that are closely linked to adherens and tight junctions. Members of this family (including AMOT, AMOTL1, and AMOTL2) possess conserved domains such as coiled-coil structures and C-terminal PDZ-binding motifs, which allow them to directly interact with the WW domains of YAP/TAZ (Zhao et al., 2011; Wang et al., 2011). This physical association anchors YAP/TAZ in the cytoplasm or at cell junctions, thereby preventing their nuclear entry and inhibiting their function as transcriptional co-activators. In addition, AMOT proteins can function as scaffolds to facilitate the activation of LATS1/2 kinases, enhancing the phosphorylation of YAP/TAZ and further promoting their binding to 14-3-3 proteins (Zhao et al., 2011). Experimental data indicate that when the expression or function of AMOT family proteins is downregulated, YAP/TAZ become dephosphorylated more readily and translocate to the nucleus, where they activate pro-proliferative and anti-apoptotic genes, thereby driving tumorigenesis and progression (Paramasivam et al., 2011).

In head and neck cancer and other epithelial-derived solid tumors, aberrant regulation of the AMOT family proteins is considered a major factor contributing to the persistent activation of YAP/TAZ. For example, the SRSF3/AMOTL1 splicing axis has been reported to promote the tumorigenesis of nasopharyngeal carcinoma by regulating the nuclear translocation of YAP1 (Xu et al., 2023). In head and neck squamous cell carcinoma, reduced expression of AMOT family proteins (such as AMOTL2) decreases their recruitment and stabilization of LATS1/2 kinases, thereby enhancing YAP nuclear translocation by suppressing LATS-dependent YAP phosphorylation (Zou et al., 2024), which is closely associated with YAP/TAZ nuclear accumulation and activation of oncogenic genes (An et al., 2023). Moreover, in other cancers such as breast and liver cancer, studies have demonstrated that the loss or degradation of AMOT family proteins correlates with aberrant activation of YAP/TAZ, thereby promoting tumor progression (Zhao et al., 2011; Paramasivam et al., 2011).

### 3.4 Non-classical Hippo pathway phosphorylation adaptor protein components

Upon activation of the Hippo pathway, LATS1/2 kinases phosphorylate key residues on YAP/TAZ (for instance, Ser127 on YAP and the corresponding site on TAZ), generating binding sites recognized by 14-3-3 proteins (An et al., 2023; Chan et al., 2011). The binding of 14-3-3 proteins not only masks the nuclear localization signals of YAP/TAZ but also promotes their stable retention in the cytoplasm, thereby preventing their nuclear translocation and transcriptional co-activation function. Further studies have revealed that this interaction also renders YAP/TAZ more susceptible to ubiquitination and degradation mediated by SCFβ-TrCP, thus exerting a negative regulatory effect on cell proliferation and survival (An et al., 2023; Chan et al., 2011).

14-3-3 proteins play crucial roles in the initiation, progression, and treatment of head and neck cancer. Among them, 14-3-3σ and 14-3-3ζ have been the focus of extensive research. 14-3-3σ regulates the chemotherapeutic sensitivity of tongue cancer through the GSK3β/β-catenin/ZEB1 pathway, and its methylation status as well as intratumoral heterogeneity are closely associated with the development of oral cancer (Peng et al., 2015). Moreover, the expression level of 14-3-3σ may serve as a potential biomarker for predicting the initial response of head and neck squamous cell carcinoma (HNSCC) to chemoradiotherapy (Huang et al., 2020). In addition, 14-3-3ζ is widely aberrantly expressed in various head and neck cancers, including oral and laryngeal cancers, and is involved in regulating cancer cell apoptosis, senescence, and drug resistance (Matta et al., 2012). Abnormal expression and impaired function of 14-3-3ζ in some head and neck cancer cells weaken its ability to sequester phosphorylated YAP/TAZ, thus facilitating their nuclear translocation and promoting cell proliferation, invasion, and chemoresistance (Macha et al., 2010). Studies have shown that guggulsterone can induce apoptosis in head and neck cancer cells by targeting 14-3-3ζ, and that siRNA-mediated downregulation of 14-3-3ζ enhances the efficacy of chemotherapeutic agents (Macha et al., 2010). Moreover, the protein interaction network of 14-3-3ζ underscores its key role in oral cancer (Matta et al., 2016), while the loss of USP18 suppress lung cancer metastasis by reducing the stability of 14-3-3ζ (Chen et al., 2022).

### 3.5 Non-classical Hippo pathway tumor suppressors

In the non-classical Hippo pathway, several tumor suppressors play critical roles in maintaining cellular homeostasis by modulating cell proliferation, apoptosis, and polarity. These factors ensure that oncogenic signals remain in check, and their inactivation or loss often contributes to uncontrolled cell growth and tumor progression.

#### 3.5.1 NF2 (Merlin)

NF2, also known as Merlin, is a key upstream tumor suppressor within the non-classical Hippo pathway. Its primary functions include the regulation of cell proliferation, apoptosis, and the maintenance of cell polarity (Harvey et al., 2003). NF2 forms complexes with molecules such as WWC1 and FRMD6, thereby promoting the activation of MST1/2 kinases. This activation subsequently enhances the phosphorylation of YAP/TAZ by LATS1/2, leading to the cytoplasmic retention of these pro-proliferative transcriptional co-activators and ultimately inhibiting aberrant tumor cell growth (Hong et al., 2020; Wu et al., 2024). Moreover, as a member of the ERM protein family, NF2 modulates the functions of E-cadherin, receptor tyrosine kinases (RTKs), and β-catenin, integrating extracellular mechanical cues and cytoskeletal signals to maintain cell–cell adhesion and polarity stability (Cao et al., 2020; Nita and Moroishi, 2024). In head and neck cancer, mutations or reduced expression of the NF2 gene often result in abnormal nuclear translocation of YAP/TAZ, thereby activating oncogenic gene expression and correlating with increased tumor invasiveness and poor patient prognosis (Nouri et al., 2024).

#### 3.5.2 RASSF family members

Members of the RASSF family (such as RASSF1A and RASSF5/NORE1A) also play important tumor suppressive roles in the non-classical Hippo pathway. RASSF1A directly interacts with MST1/2 kinases, preventing dephosphorylation by the PP2A phosphatase, thus maintaining MST1/2 activity and promoting the propagation of apoptotic signals (Bae et al., 2017; Oh et al., 2006). Additionally, RASSF1A can directly bind to microtubules, stabilizing their dynamics and thereby inhibiting cell migration and tumor metastasis (Dallol et al., 2004). RASSF5 (NORE1A), acting downstream of Ras, activates MST1/2 to induce cell cycle arrest and Fas-dependent apoptosis (Shivakumar et al., 2002; Graves et al., 2001). Furthermore, through its conserved SARAH domain, RASSF family members form heterodimers with MST1/2 to modulate other non-classical apoptotic signaling pathways, such as the JNK/SAPK pathway, which further suppresses tumor cell proliferation (Hwang et al., 2007). In head and neck cancer, RASSF family members are frequently inactivated due to promoter methylation or gene mutations, a loss of function that is closely associated with decreased MST1/2 activity and aberrant activation of YAP/TAZ, ultimately leading to uncontrolled cell proliferation and tumor metastasis (Dhanaraman et al., 2020; Iwasa et al., 2015).

The non-canonical Hippo pathway orchestrates head and neck carcinogenesis through a diverse array of molecular components, as discussed above (Figure 1). Core kinases (TAO, MAP4K, NDR1/2), polarity regulators (CRUMBS/SCRIBBLE), junction proteins (AMOT family), and tumor suppressors (NF2, RASSF family) collectively maintain cellular homeostasis via phosphorylation-dependent signaling and cytoskeletal interactions. Meanwhile, post-translational modifications—ubiquitination, acetylation, and SUMOylation—fine-tune effector stability and activity, with dysregulation driving YAP/TAZ hyperactivation and tumor progression (Table 1).

**TABLE 1:** Functional classification of non-canonical hippo pathway components in head and neck cancer.

Category	Key molecules	Mechanistic insights	Clinical relevance in HNC	Refs
Core Kinases	TAO kinases (TAOK1/2/3)	Phosphorylates/activates MST1/2 to initiate LATS1/2-mediated YAP/TAZ cytoplasmic retention	Dysregulation enhances YAP/TAZ nuclear translocation, driving tumor proliferation	Fang et al. (2020), Kapfhamer et al. (2012), Boggiano et al. (2011)
	MAP4K family kinases	Directly phosphorylates LATS1/2 or modulates YAP/TAZ via non-canonical AMPK/mTOR pathways	MAP4K4 activates JNK signaling to promote migration and chemoresistance in oral cancers	Meng et al. (2015), Li et al. (2015)
Polarity Regulators	CRUMBS/SCRIBBLE	Maintains apical-basal polarity via membrane architecture regulation, limiting YAP/TAZ nuclear access	Polarity disruption facilitates YAP/TAZ-driven tumorigenesis	Muthuswamy and Xue (2012), Stephens et al. (2018)
Junction Proteins	AMOT family (AMOT/L1/L2)	Binds YAP/TAZ WW domains via PPxY motifs, sequesters in cytoplasm, and enhances LATS1/2 activity	Downregulation correlates with increased YAP/TAZ nuclear accumulation and invasion	Zhao et al. (2011), Wang et al. (2011), Paramasivam et al. (2011)
Phospho-adaptors	14-3-3 proteins	Binds p-Ser127 on YAP/TAZ to mask nuclear localization signals and promote degradation	14-3-3ζ dysfunction accelerates YAP/TAZ-mediated progression and therapy resistance	Chan et al. (2011), Macha et al. (2010)
Tumor Suppressors	NF2 (Merlin)	Activates MST1/2 through WWC1/FRMD6 complexes to enhance YAP/TAZ phosphorylation	Mutations/loss correlate with YAP/TAZ activation and poor prognosis	Harvey et al. (2003), Hong et al. (2020), Wu et al. (2024)
	RASSF family (RASSF1A/5)	RASSF1A stabilizes MST1/2; RASSF5 induces apoptosis via Ras-MST1/2 signaling	RASSF1A methylation/mutation induces YAP/TAZ activation, promoting metastasis	Bae et al. (2017), Oh et al. (2006), Dallol et al. (2004), Dhanaraman et al. (2020), IWASA et al. (2015)
PTM Regulators	MST1/2 (Ubiquitination)	Smurf1-mediated ubiquitination degrades MST1/2, suppressing pathway activity	Inactivation drives YAP/TAZ activation and tumor progression	Del et al. (2010), Xu et al. (2023b), Santos-de-frutos et al. (2019)
	ΔNp63 (Ubiquitination/Acetylation)	WWP1-mediated degradation and HDAC2-modulated acetylation regulate epithelial differentiation	Aberrant degradation promotes invasion and metastasis	Low-calle et al. (2023), Wang et al. (2019), Gallant-behm and Espinosa (2013)
Crosstalk Pathways	Wnt (VGLL4)	Competes with YAP/TAZ for TEAD binding, antagonizing Wnt/β-catenin signaling	VGLL4 downregulation enables YAP/TAZ-β-catenin co-activation in tumorigenesis	Jiao et al. (2017)
	NF-κB/ER pathways	NF-κB-YAP/TAZ interaction drives inflammation; ERα activates PI3K/AKT to inhibit LATS1/2; ERβ/GPER modulates immune escape	Potential therapeutic targets requiring mechanistic clarification in HNC	Senftleben et al. (2001), Chan et al. (2005)

### 3.6 Hippo pathway dysregulation drives HNSCC initiation and therapy resistance

Genomic analyses of HNSCC from TCGA have revealed frequent inactivating mutations of FAT1 (29%), amplifications of WWTR1 (TAZ, 14%) and YAP1 (8%), all of which associate with persistent YAP/TAZ activation and poor clinical outcome (Chowl, 2020; Moya and Halder, 2019; Dey et al., 2020; Fang et al., 2020; Chen et al., 2019)]. Transcriptomic profiling corroborates these findings, showing that elevated YAP1 and WWTR1 mRNA levels in tumor specimens correlate with advanced clinical stage, higher metastatic risk, and shorter overall survival (Moya and Halder, 2019) (Hermann et al., 2021); immunohistochemistry further confirms that nuclear YAP positivity is enriched in late-stage, chemoresistant cases (Hermann et al., 2021). Functionally, overexpression of YAP1 in HNSCC cell lines (FaDu, SCC-25) markedly enhances proliferation, migration, and cisplatin resistance, whereas knockdown of YAP1 or TAZ restores drug sensitivity and induces apoptosis (Moya and Halder, 2019; Boggiano et al., 2011) (Moya and Halder, 2019; Boggiano et al., 2011). In murine xenograft models, YAP1 depletion in FaDu cells slows tumor growth by ∼50% and reduces pulmonary metastases, and treatment with Verteporfin (a YAP–TEAD inhibitor) further suppresses tumor progression and diminishes resistant lesions (Boggiano et al., 2011). Moreover, hyperactive EGFR signaling drives MOB1 tyrosine phosphorylation to inhibit LATS1/2, thereby promoting YAP nuclear accumulation and induction of resistance-related genes; combined inhibition of EGFR and YAP signaling synergistically attenuates tumor growth and reverses therapeutic resistance (Fu et al., 2022).

## 4 Targeting post-translational modifications of Hippo pathway proteins

### 4.1 Ubiquitination regulation of MST1/2

As key kinases in the non-classical Hippo pathway, the activity of MST1/2 is critical for maintaining the balance between cell proliferation and apoptosis. Studies have demonstrated that the E3 ubiquitin ligase Smurf1 can ubiquitinate MST1/2 at the K285/K282 residues, thereby promoting their degradation and diminishing their tumor-suppressive functions (Xu Y. et al., 2023). In addition, RASSF1A can indirectly stabilize MST1/2 activity by preventing PP2A-mediated dephosphorylation; however, if RASSF1A itself is degraded via ubiquitination, the loss of MST1/2 signaling is further exacerbated (Del et al., 2010). In head and neck cancer, reduced MST1/2 activity is closely associated with sustained activation of YAP/TAZ, which in turn drives cell proliferation and invasion (Shin and Kim, 2020; Santos-de-frutos et al., 2019; Ando et al., 2022).

### 4.2 Post-translational modifications of the TEAD family

TEAD transcription factors, as key downstream effectors of the Hippo pathway, depend not only on their interaction with YAP/TAZ but also on post-translational modifications for proper function. Studies have shown that palmitoylation of TEAD proteins occurs on conserved cysteine residues, enhancing the stability of TEAD-YAP/TAZ binding and thereby promoting the transcription of oncogenic genes (Lin et al., 2017). In parallel, ubiquitination mediated by E3 ubiquitin ligases (such as RNF146) may influence TEAD stability and nuclear localization, thereby modulating its transcriptional activity (Landin-Malt et al., 2016). Abnormal activation of TEAD function in head and neck cancer is closely linked to YAP/TAZ-driven proliferation and migration (Zhao et al., 2007; Zhao et al., 2008; Huh et al., 2019; Noland et al., 2016).

### 4.3 Ubiquitination regulation of RASSF1A

As a tumor suppressor within the non-classical Hippo pathway, RASSF1A maintains MST1/2 kinase activity through interaction via its SARAH domain (Guo et al., 2011). However, RASSF1A can be ubiquitinated by ITCH and the CUL4A-DDB1 complex, leading to its degradation. This degradation releases its positive regulatory effect on MST1/2, allowing YAP/TAZ to escape inhibition (García-gutiérrez et al., 2020; Jiang et al., 2011). Promoter methylation-mediated inactivation of RASSF1A is common, and aberrations in the ubiquitination pathway further disrupt Hippo signaling, thereby promoting tumor development (Del et al., 2010; Rabizadeh et al., 2004).

### 4.4 Ubiquitination regulation of Beclin 1

Beclin 1, an important regulator of autophagy, is subject to control by ubiquitination. For instance, TRAF6-mediated K63-linked ubiquitination promotes Beclin 1 oligomerization, which activates the autophagy-related protein ULK1 and enhances autophagic activity (Zalckvar et al., 2009; Shi and Kehrl, 2010). Conversely, the deubiquitinating enzymes USP10 and USP13 reduce Beclin 1 ubiquitination levels, thereby inhibiting autophagy (Liu et al., 2011). In certain tumors, including head and neck cancer, dysregulated autophagy is closely associated with cancer cell survival, migration, and drug resistance, suggesting that abnormalities in the ubiquitination status of Beclin 1 may significantly influence cancer cell fate (Li et al., 2017; Ashkenazi et al., 2017).

### 4.5 Ubiquitination regulation of MOB proteins

MOB1, a coactivator in the Hippo pathway, promotes the phosphorylation of YAP/TAZ through interactions with MST1/2 and LATS1/2. Its stability is also regulated by ubiquitination (Ando et al., 2021). E3 ubiquitin ligases, such as praja2, can ubiquitinate MOB1, thereby reducing its stability within the cell and indirectly weakening the inhibitory effect of the Hippo pathway on YAP/TAZ. This regulatory mechanism potentially contributes to the aberrant activation of YAP/TAZ observed in head and neck cancer (Liu and Deng, 2019; Yang et al., 2024).

## 5 Cross-regulation between the Hippo pathway and other signaling pathways in head and neck cancer

In recent years, accumulating evidence has revealed that the transcriptional co-regulators of the non-classical Hippo signaling pathway encompass not only the classical YAP/TAZ-TEAD regulatory mode but also integrate inputs from non-classical Wnt, NF-κB, and estrogen receptor (ER) signaling pathways. These pathways regulate the activity of YAP, TAZ, and other related transcription factors through their distinct molecular mechanisms, thereby influencing cell proliferation, differentiation, apoptosis, and the formation of the tumor microenvironment. Consequently, they play critical roles in the initiation and progression of solid tumors, including head and neck cancer.

### 5.1 Crosstalk between the Wnt signaling pathway and the Hippo pathway

The Wnt signaling pathway (e.g., Wnt5a, Wnt4) activates downstream kinases such as RhoA/ROCK via its receptors (e.g., ROR2 and FZD2), thereby regulating cytoskeletal reorganization and cell polarity. This process significantly influences the activation status of the Hippo pathway (Wang and Martin, 2017). Studies have shown that activation of Wnt signaling can inhibit LATS1/2 kinase activity, resulting in decreased phosphorylation of YAP/TAZ. The ensuing dephosphorylation enables their nuclear translocation and enhances their binding to TEAD, which in turn initiates the transcription of pro-proliferative genes (Kriz and Korinek, 2018; Wei et al., 2018). VGLL4, a key transcriptional co-regulator within the non-classical Hippo pathway, competitively binds TEAD family proteins via its two conserved Tondu domains, thereby blocking the interaction between YAP/TAZ and TEAD. This interference not only prevents YAP/TAZ nuclear entry but also diminishes TEAD-mediated transcriptional activity, effectively maintaining the balance between cell proliferation and apoptosis. Moreover, VGLL4 negatively regulates TCF4-mediated transcription under the Wnt/β-catenin pathway, thereby achieving coordinated cross-pathway regulation (Jiao et al., 2017). In head and neck cancer, this Hippo-Wnt crosstalk is particularly critical; downregulation of VGLL4 is common in certain head and neck squamous cell carcinomas, leading to the co-activation of YAP/TAZ and β-catenin, which in turn promotes cancer cell proliferation and invasion.

### 5.2 Crosstalk between the NF-κB pathway and the Hippo pathway

The NF-κB signaling pathway is a key regulator of inflammatory responses, cell survival, and immune modulation, with its activation primarily dependent on IκB degradation mediated by the IKK complex (Tam et al., 2012). Studies have indicated that YAP/TAZ are not only downstream effectors of the Hippo pathway but can also modulate NF-κB activity through interactions with the IKK complex (Senftleben et al., 2001; Hayden and Ghosh, 2008). Under certain inflammatory and stress conditions, activation of the NF-κB pathway can upregulate key proteins associated with the Hippo pathway, such as MST1/2, thereby forming a negative feedback regulatory loop. Conversely, inactivation of the Hippo pathway (resulting in YAP/TAZ nuclear translocation) can enhance the expression of NF-κB target genes (e.g., pro-inflammatory factors IL-6 and TNF-α), thereby promoting the establishment of the tumor microenvironment (Chan et al., 2005; Hayden and Ghosh, 2008). In head and neck cancer, where chronic inflammation is common, the crosstalk between NF-κB and the Hippo pathway contributes to cancer cell proliferation, migration, and immune evasion, providing a molecular basis for tumor progression and drug resistance.

### 5.3 Crosstalk between the estrogen receptor (ER) pathway and the Hippo pathway

The estrogen receptor signaling pathway primarily regulates cell proliferation, differentiation, and survival through ERα and related receptors (e.g., GPER). In certain breast cancer studies, the interaction between ER signaling and the Hippo pathway has been preliminarily elucidated: activation of ERα can indirectly inhibit LATS1/2 activity via upregulation of the PI3K/AKT pathway, thereby promoting the dephosphorylation and nuclear accumulation of YAP/TAZ (Klinge, 2001; Filardo et al., 2000). Moreover, non-classical estrogen signaling mediated by GPER has been found to activate YAP/TAZ, and by enhancing the expression of downstream effector genes such as CTGF, it promotes cell migration and invasion (Gutierrez et al., 2005; Kim et al., 2020; Chimento et al., 2022). Although head and neck cancer is not typically classified as a hormone-dependent tumor, some studies suggest that ERβ and GPER may also participate in the regulation of the Hippo pathway in head and neck squamous cell carcinoma, thereby affecting tumor growth and immune evasion (Cheng et al., 2011; Wu and Yang, 2018). This cross-regulation mechanism provides a novel avenue for exploring potential therapeutic targets within the non-classical ER-Hippo signaling axis in head and neck cancer.

### 5.4 Crosstalk between the ΔNp63 and the Hippo pathway

ΔNp63, a transcription factor closely related to epithelial differentiation, is regulated at the level of protein stability by both ubiquitination and deacetylation. The E3 ubiquitin ligase (e.g., WWP1) mediates the ubiquitination of ΔNp63α, promoting its proteasomal degradation and thereby altering its regulatory role in cell differentiation and migration (Wang et al., 2019). Concurrently, deacetylation mediated by HDAC2 enhances the transcriptional repressive function of ΔNp63α, consequently affecting cell fate decisions (Gallant-behm and Espinosa, 2013; Qian et al., 2011). In head and neck squamous cell carcinoma, abnormal degradation or altered stability of ΔNp63 disrupts normal epithelial differentiation, which promotes cancer cell invasion and metastasis (Low-Calle et al., 2023).

### 5.5 Integrated impact of cross-regulation in head and neck cancer and its clinical significance

In head and neck cancer, the cross-regulation between the non-classical Wnt, NF-κB, and ER signaling pathways with the Hippo pathway forms a complex network that plays a crucial role in modulating YAP/TAZ activity. VGLL4, an important downstream regulator of the Hippo pathway, inhibits YAP/TAZ transcriptional activity through competitive binding with TEAD and integrates Wnt signaling to suppress TCF4-driven gene expression, thereby exerting inhibitory effects on cell proliferation, invasion, and immune evasion (Wang and Martin, 2017; Wei et al., 2018; Lin et al., 2017). Activation of the NF-κB pathway upregulates pro-inflammatory and anti-apoptotic genes, synergizing with YAP/TAZ to drive tumor progression, while the ER signaling pathway modulates YAP/TAZ nuclear translocation and stability via GPER and ERα. The integrated effects of these pathways result in the pronounced abnormal activation of YAP/TAZ in head and neck cancer, which is closely associated with poor prognosis, drug resistance, and immune evasion. Future therapeutic strategies targeting this multi-pathway regulatory network—such as combined targeting of YAP/TAZ with NF-κB, Wnt, or ER signaling—hold promise for improving treatment outcomes in head and neck cancer patients (Jiao et al., 2017; Wu and Yang, 2018; Yu et al., 2019).

## 6 Clinical significance and therapeutic strategies of the non-classical Hippo pathway in head and neck cancer

The non-classical Hippo pathway plays a crucial role in the initiation, progression, and drug resistance of head and neck cancer by regulating multiple signaling cascades and their downstream transcriptional co-regulators. In recent years, numerous studies have revealed that abnormal expression of core effectors—such as YAP/TAZ and MOB1—as well as upstream regulators (e.g., MST1/2, RASSF1A, NF2) is closely associated with patient prognosis, tumor invasiveness, and therapeutic response. These findings underscore the potential of these molecules as biomarkers and therapeutic targets.

### 6.1 Diagnostic and prognostic value of the non-classical Hippo pathway in head and neck cancer

Accumulating evidence indicates that core components of the non-classical Hippo pathway serve as both early diagnostic and prognostic biomarkers in head and neck squamous cell carcinoma (HNSCC). Aberrant nuclear localization of YAP/TAZ—often driven by high-frequency genomic events such as FAT1 loss, WWTR1 amplification and YAP1 amplification—correlates with increased tumor proliferation, metastasis, chemoresistance and significantly shorter overall and progression-free survival (Faraji et al., 2022; Zhao et al., 2007; Yu et al., 2015). Concurrently, reduced phosphorylation of MOB1 marks Hippo pathway dysfunction and portends poorer outcomes (Tang et al., 2023; Delgado et al., 2020). Elevated ΔNp63 expression, regulated via non-canonical interactions with polarity and junctional proteins (e.g., AMOT, ZO-1/2), associates with cancer stem-like properties, EMT and drug resistance, and the ΔNp63/YAP ratio enables molecular subtyping and risk stratification (Low-Calle et al., 2023; Fallahi et al., 2016). Early detection strategies encompass molecular assays—single-cell RNA-seq to resolve intratumoral heterogeneity and qPCR/ddPCR or targeted NGS for FAT1, YAP1 and WWTR1 alterations (Faraji et al., 2022; Ando et al., 2022)—as well as proteomic approaches, including iTRAQ mass spectrometry and phosphoproteomic profiling of YAP/TAZ activity, complemented by IHC scoring of nuclear localization (Faraji et al., 2022; Santos-de-frutos et al., 2019). Liquid biopsy of circulating miRNAs, lncRNAs and Hippo-related exosomes offers a minimally invasive readout of pathway activation (Tu et al., 2020), while optical modalities such as surface-enhanced Raman spectroscopy and narrow-band imaging achieve high sensitivity and specificity for early HNSCC detection (Shin and Kim, 2020). Finally, YAP/TAZ-mediated upregulation of immune checkpoint ligand PD-L1 and chemokines (CXCL5, CCL2), together with hypoxia-responsive metabolic markers (SERPINE1, CA9), provide additional immune and metabolic biomarkers for early diagnostic screening (Tu et al., 2020) (Tang et al., 2023; Azad et al., 2020). Together, these multi-modal assays promise to enhance early diagnosis and prognostic assessment in head and neck cancer.

### 6.2 Targeted therapeutic strategies for the non-classical Hippo pathway

Therapeutic strategies targeting the non-classical Hippo pathway in head and neck cancer involve both direct and indirect approaches. Direct inhibition strategies focus on disrupting the YAP/TAZ-TEAD interaction using small molecules or peptide-based agents, while indirect strategies aim to restore upstream regulatory mechanisms. Moreover, combination therapies that integrate Hippo pathway inhibitors with immunotherapy or other targeted agents hold promise for overcoming resistance and enhancing clinical efficacy.

#### 6.2.1 Direct inhibition using small molecule or peptide-based agents

Direct inhibition of the YAP/TAZ-TEAD complex is currently a research hotspot. For instance, pan-TEAD inhibitors such as GNE-7883 block the interaction between YAP/TAZ and TEAD, thereby significantly inhibiting tumor growth and reversing resistance to KRAS inhibitors (Thrash and Pendergast, 2023; Thompson, 2020). Additionally, peptide-based drugs designed on the basis of the α-helical structure of VGLL4, which mimic its competitive binding to TEAD, have shown potential in blocking YAP/TAZ-driven transcriptional activation and reducing tumor cell proliferation and migration (Kim et al., 2018).

Small‐molecule pan‐TEAD inhibitors such as GNE-7883 exhibit a short plasma half‐life (∼42–4 h), high plasma‐protein binding (>95%) and moderate oral bioavailability (∼40%), with preclinical toxicology showing no significant weight loss or biochemical abnormalities below the maximum tolerated dose. In first‐in‐human studies, the TEAD inhibitors IK-930 and VT-3989 demonstrated dose‐proportional increases in C_max and AUC, alongside manageable grade I–II adverse events (primarily mild gastrointestinal disturbances and thrombocytopenia), indicating predictable pharmacokinetics and an acceptable safety profile (Zagiel et al., 2022). Complementary to small molecules, α-helical peptides modeled on Vgll4 are delivered via PEG–PLA nanoliposomes to extend systemic exposure (circulation half‐life increased from 0.6 h to 3.2 h) and boost tumor accumulation by 2.5-fold in murine HNSCC models, while markedly reducing hepatotoxicity and nephrotoxicity (Liu et al., 2019). Advanced delivery platforms—including peptide–polysaccharide conjugates (e.g., peptide–PEG–cholesterol) and lipid nanoparticles—are under development to stabilize these peptides and enhance cellular uptake for clinical application. Given TEAD’s physiological roles in cardiac and hepatic tissue, careful toxicity assessment remains essential: murine multi-dose studies at ≤10 mg/kg/day maintained normal ALT/AST and CK-MB levels, and early clinical data report <5% incidence of ECG abnormalities at therapeutic IK-930 exposures, supporting a favorable therapeutic window (Dey et al., 2020). Future strategies aim to exploit tumor microenvironment–responsive carriers (e.g., pH- or MMP-triggered nanoparticles) for on-site drug release, antibody-functionalized systems targeting EGFR or CD44 to achieve HNSCC specificity, and PLGA-based sustained‐release formulations to extend dosing intervals and improve patient compliance.

#### 6.2.2 Indirect modulation strategies

Restoration of Hippo pathway function via upstream receptor modulation represents an important indirect approach. For example, EGFR activation, which is commonly observed in head and neck cancer, promotes tyrosine phosphorylation of MOB1 and subsequently inhibits LATS1/2 activity. Therefore, EGFR-targeted drugs (e.g., osimertinib) can indirectly restore LATS1/2-mediated inhibition of YAP/TAZ (Nottingham et al., 2014; Guerrero-preston et al., 2017). Moreover, modulation of GPCR signaling (e.g., LPA receptors) offers another intervention target, as blocking these signals can reduce YAP/TAZ nuclear translocation (Yu et al., 2012; Chen and Jin, 2023). In addition, metabolic and microenvironmental interventions—such as the use of antioxidants (e.g., NAC) or AMPK activators to regulate MOB1 acetylation—also hold promise for exerting antitumor effects through indirect modulation of the Hippo pathway (Elbediwy et al., 2016; Heidary et al., 2017; Jin J. et al., 2022; Liu et al., 2023).

#### 6.2.3 Combination therapies and reversal of drug resistance

Given that head and neck cancer is often accompanied by immune evasion and drug resistance, combination therapeutic strategies are gaining increasing attention. Studies have shown that YAP/TAZ activation upregulates PD-L1 expression, leading to tumor immune suppression; hence, the combination of Hippo pathway inhibitors (e.g., verteporfin) with immune checkpoint inhibitors (e.g., anti-PD-1/PD-L1 antibodies) can synergistically enhance antitumor immunity (Janse van rensburg et al., 2018; Zeng and Dong, 2021; Sun et al., 2023). Furthermore, in resistant cancer cells, activation of a non-classical YAP1-TEAD2 axis mediated by HER4 is closely associated with drug resistance, and combining HER inhibitors (e.g., lapatinib) may reverse resistance to trastuzumab (González-Alonso et al., 2020; Mehra et al., 2011). These strategies offer new perspectives for overcoming resistance associated with monotherapy.

### 6.3 Multi-omics integration for biomarker discovery and therapeutic optimization

Moreover, with the rapid advancement of precision oncology, multi-omics approaches have become indispensable for unraveling how Hippo pathway dysregulation drives tumor heterogeneity and therapy response. Transcriptomic profiling of YAP/TAZ and their downstream effectors—such as CTGF and CYR61—allows quantitative assessment of pathway activation and can predict sensitivity to YAP–TEAD inhibitors like verteporfin in breast cancer and glioblastoma models (Zhao et al., 2023; Lv and Zhou, 2020). Notably, transcriptome analyses have identified malignant subgroups in low-grade glioma and melanoma characterized by elevated YAP expression, which are associated with poorer prognosis (FU et al., 2022; Zhao et al., 2023). Proteomic studies, particularly phosphoproteomics, provide a direct measure of YAP/TAZ activation status and nuclear localization, thereby forecasting biological responses to Hippo pathway inhibitors and revealing bypass resistance mechanisms such as EGFR signaling (Lv and Zhou, 2020). At the epigenomic level, DNA methylation–mediated silencing of tumor suppressors like RASSF1A correlates closely with YAP/TAZ hyperactivation, while non-coding RNAs (e.g., MIR100HG, GASS) modulate YAP’s nuclear import and transcriptional activity, offering candidate markers for resistance prediction and novel therapeutic targets (Tu et al., 2020; Sun and Chi, 2021).

### 6.4 Current clinical trials and future perspectives

Currently, targeted drugs against core components of the non-classical Hippo pathway—especially the YAP/TAZ-TEAD complex—have entered clinical trial phases. For instance, TEAD inhibitors such as IK-930 (NCT05228015) and VT-3989 (NCT04665206) are in Phase I/II clinical trials, with indications that include head and neck cancer (Ando et al., 2022; Thompson, 2020; Zeng and Dong, 2021). Future studies will further integrate multi-omics data to explore precision biomarkers—such as YAP/TAZ activity scores based on CTGF and CYR61 expression—to optimize therapeutic regimens (Shin and Kim, 2020). At the same time, novel drug delivery systems (e.g., nanoparticle-mediated siRNA delivery) have shown promising preclinical results, potentially addressing issues related to off-target effects and drug resistance of small molecule agents (Kim et al., 2018; Wu et al., 2021; Rybarczyk et al., 2017). Overall, as our understanding of the regulatory mechanisms governing the non-classical Hippo pathway advances, targeted therapies based on this pathway are expected to achieve clinical translation in head and neck cancer and other solid tumors, ultimately improving patient prognosis and survival (Dey et al., 2020; Chen L. et al., 2010).

## 7 Discussion

Emerging research on the non-canonical Hippo pathway has elucidated its crucial regulatory roles in cellular proliferation, apoptosis, migration, and immune modulation, particularly in the pathogenesis of head and neck cancers. However, several critical issues remain to be addressed. First, the intricate regulatory network involving multiple signaling molecules (e.g., MST1/2, RASSF family members, and NF2) and their crosstalk with other pathways (including Wnt, NF-κB, and estrogen receptor signaling) remains incompletely understood (Li et al., 2023). While current evidence demonstrates the essential regulatory functions of Smurf1-mediated MST1/2 ubiquitination and ITCH/CUL4A-DDB1-dependent RASSF1A degradation in Hippo pathway modulation (Guo et al., 2011), the synergistic interplay between these ubiquitination modifications and other post-translational modifications (e.g., acetylation and palmitoylation) in determining YAP/TAZ nucleocytoplasmic shuttling and subsequent transcriptional activation requires more systematic investigation.

Second, pathway heterogeneity across tumor types warrants special attention. Accumulating data indicate distinct YAP/TAZ activation patterns in head and neck squamous cell carcinoma (HNSCC) subtypes, with frequent nuclear accumulation of YAP/TAZ correlating with poor prognosis and notably higher activity observed in HPV-negative cases (Faraji et al., 2022; Zamuner et al., 2024). Furthermore, significant variations in expression profiles and functional characteristics of key components (including VGLL4, NF2, and RASSF family members) have been documented across various malignancies (e.g., breast, hepatic, and colorectal cancers) (Zhai et al., 2023; Bevanda et al., 2025). These molecular divergences, while providing potential biomarkers for patient stratification and personalized therapy, complicate comprehensive pathway analysis and therapeutic targeting.

Recent advancements in detection technologies and targeted therapies present both opportunities and challenges. Novel approaches integrating molecular imaging, liquid biopsy, and multi-omics analysis show promise for non-invasive monitoring of pathway activity, though current limitations in sensitivity (particularly for low-abundance biomarkers) and standardization hinder clinical implementation (Jin C. et al., 2022; Lone et al., 2022; He et al., 2023). Therapeutically, emerging agents such as TEAD inhibitors (IK-930, VT-3989) and VGLL4-based peptide drugs demonstrate encouraging preclinical efficacy, yet face obstacles including compensatory pathway activation and acquired resistance mechanisms (Zhu et al., 2021). Notably, combination strategies with immune checkpoint inhibitors reveal synergistic potential, but optimal drug pairing, delivery system optimization, and toxicity management require further elucidation.

In conclusion, while the non-canonical Hippo pathway emerges as a promising therapeutic target in head and neck oncology, overcoming current challenges—including pathway complexity, tumor subtype heterogeneity, and technical limitations in detection/therapeutic modalities—will require coordinated efforts in fundamental research and clinical translation. Addressing these issues through integrated multidisciplinary approaches may ultimately advance precision medicine paradigms and improve patient outcomes.

## 8 Conclusion

This review synthesizes current understanding of the non-canonical Hippo pathway’s multilayer regulatory mechanisms in head and neck cancers, highlighting its complex signaling network comprising core kinase components (e.g., TAO, MAP4K, and NDR1/2 kinases), cell polarity regulators (CRUMBS/SCRIBBLE), cell junction modulators (AMOT family), and 14–3-3 scaffold proteins. Notably, tumor suppressors like NF2 (Merlin) and RASSF family members exert anti-cancer effects through cytoskeletal interactions and membrane protein regulation, essential for cellular homeostasis maintenance.

Post-translational modifications (ubiquitination, acetylation, SUMOylation) of key components—including MST1/2, ΔNp63, TEADs, and MOB proteins—precisely orchestrate YAP/TAZ nucleocytoplasmic dynamics, thereby modulating pro-tumorigenic processes such as proliferation, metastasis, therapy resistance, and immune evasion. Clinically, aberrant YAP/TAZ activation emerges as a biomarker with diagnostic, prognostic, and therapeutic relevance in head and neck oncology.

The pathway’s crosstalk with Wnt, NF-κB, and estrogen receptor signaling forms a dynamic regulatory network, offering rationale for multi-targeted therapies. Emerging strategies targeting YAP/TAZ-TEAD interactions (small-molecule/peptide inhibitors), upstream receptors, and metabolic regulators, particularly when combined with immune checkpoint blockade, show preclinical promise. However, pathway complexity and compensatory resistance mechanisms demand deeper mechanistic exploration.

Future investigations should focus on deciphering the context-dependent crosstalk between non-canonical Hippo components and their interacting partners, particularly under therapeutic stress conditions. This requires developing advanced detection platforms capable of real-time monitoring of pathway dynamics in tumor microenvironments, while simultaneously addressing technical limitations in sensitivity and standardization. Parallel efforts must prioritize optimizing synergistic treatment regimens through rational combination of YAP/TAZ inhibitors with immune modulators or epigenetic drugs, supported by engineered delivery systems to enhance tumor specificity. Ultimately, bridging these mechanistic insights with clinical validation through biomarker-driven trials will be crucial for translating Hippo pathway targeting into durable therapeutic responses.
