# Supplementary figures and images for: Atypical Hippo signaling network: uncovering novel insights into head and neck cancer biology and advancements in precision intervention
Source: Front Cell Dev Biol. 2025 May 23;13:1610471. doi: 10.3389/fcell.2025.1610471 (PMC12141295; doi:10.3389/fcell.2025.1610471)

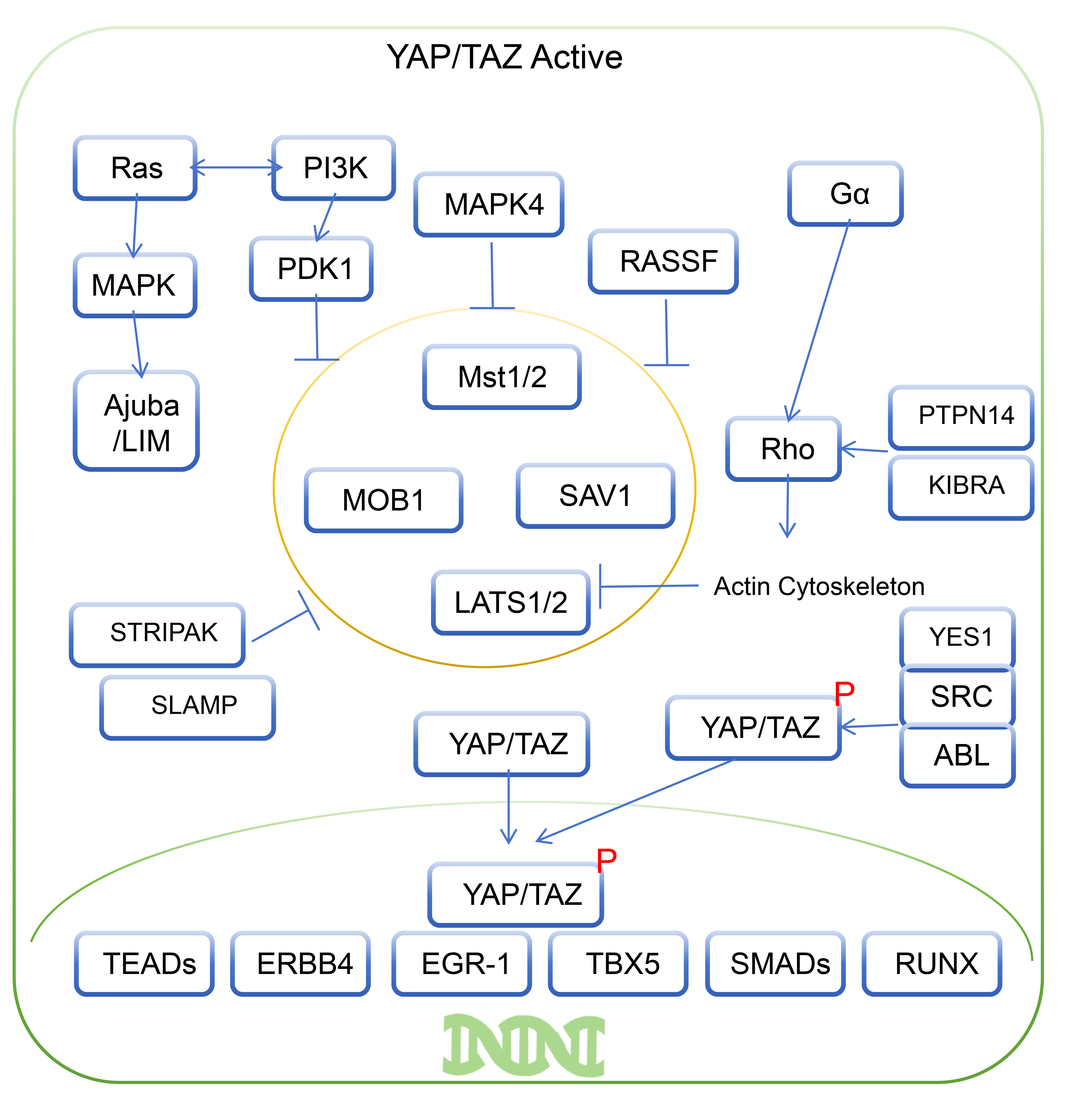

Supplement: Supplementary file 1 [file Image1.tif]
